# A narrative synthesis of factors that affect women speaking up about early warning signs and symptoms of pre-eclampsia and responses of healthcare staff

**DOI:** 10.1186/s12884-017-1245-4

**Published:** 2017-02-13

**Authors:** Wendy Carter, Debra Bick, Nicola Mackintosh, Jane Sandall

**Affiliations:** 1 King’s College London, Division of Women’s Health, Faculty of Life Sciences and Medicine, London, UK; 2 King’s College London, Florence Nightingale Faculty of Nursing and Midwifery, London, UK; 3 King’s College London, Women’s Health Academic Centre, King’s Health Partners, 10th Floor North Wing, St Thomas’ Hospital, Westminster Bridge Road, SE1 7EH London, UK

**Keywords:** Pre-eclampsia, Narrative synthesis review, Midwives, Help seeking

## Abstract

**Background:**

One of the challenges for treating pre-eclampsia and preventing further deterioration is determining how best to enable early detection. If women or their partners and families are able to raise early warnings about potential signs and symptoms of pre-eclampsia in pregnancy, birth and in the postnatal period, women may be able to receive earlier intervention to prevent severe pre-eclampsia from developing. The aim of this study was to improve understanding of factors affecting the ability of women to recognise symptoms and signs of pre-eclampsia/eclampsia and seek appropriate medical help and factors affecting health care professionals’ responses to women and their families who ‘speak up’ about early warning signs and symptoms.

**Methods:**

A narrative synthesis was conducted of evidence relevant to address the research question. The following electronic data bases were searched for qualitative studies which met inclusion criteria from January 1980 to April 2016; Medline, CINAHL, HMIC, PsycINFO, Embase, BNI, ASSIA, Scopus, Maternity and Infant Care, Web of Science, Google Scholar, Cochrane, JBI and IBSS with the support of an Information Service Consultant.

**Results:**

Following thematic analysis, three themes were identified; 1: Women’s understanding and knowledge of pre-eclampsia/eclampsia; 2: Factors affecting help seeking behaviour from perspectives of women and their families’; 3: Factors affecting staff response. There was widespread lack of knowledge and understanding of signs and symptoms of pre-eclampsia/eclampsia among women and their families, with some women not exhibiting signs and symptoms of pre-eclampsia or unable to distinguish them from ‘normal’ pregnancy changes.

**Conclusions:**

Women and their families not only need to be made aware of signs and symptoms of pre-eclampsia/eclampsia but also require information on the most effective ways to seek urgent medical assessment and care. Some women did not experience prodromal signs and symptoms, which raises concerns about how women and families can detect early onset, and is an issue which needs further exploration. There is very limited research exploring clinical staff response to women who raise concerns about their health when experiencing symptoms and signs of pre-eclampsia/eclampsia with further research needed if safety and quality of care are to be improved.

## Background

Pre-eclampsia is a major obstetric problem affecting 2–8% of pregnancies and is a leading global cause of maternal and perinatal mortality and morbidity [1]. It is a pregnancy specific disease characterised by de-novo development of concurrent hypertension and proteinuria, sometimes progressing into a multisystem disorder [1]. The potential adverse perinatal outcomes include intrauterine growth restriction, preterm birth and fetal death [2]. If untreated, pre-eclampsia can develop into eclampsia when maternal seizures develop [3]. HELLP Syndrome (Haemolysis, Elevated liver enzymes and Low Platelet) is regarded as a variant of severe pre-eclampsia and can occur in 10 – 20% of cases with severe pre-eclampsia [4].

The incidence of pre-eclampsia and eclampsia varies internationally. Eclampsia is is more common in developing countries than in high income countries [5]. For example, in Latin America and the Caribbean hypertensive disorders are responsible for around 26% of maternal deaths, whereas in Africa and Asia they contribute to around 9% of deaths [1]. The incidence of pre-eclampsia is reported to have increased in the USA, which may be related to an increased prevalence of predisposing factors such as chronic hypertension, diabetes and obesity [6].

For the purposes of this synthesis pre-eclampsia was defined as “a combination of a new hypertension and proteinuria and typically occurs after 20 weeks gestation” [7]. Severe pre-eclampsia is pre-eclampsia with severe hypertension and/or symptoms and/or biochemical and/or haematological impairment and eclampsia is defined as a convulsive condition associated with pre-eclampsia [7] which can also present for the first time in the postnatal period [8]. For purposes of brevity in the remainder of the paper the term pre-eclampsia should also be understood to include severe pre-eclampsia and eclampsia unless otherwise indicated.

One of the challenges for treating pre-eclampsia and preventing further deterioration is determining how best to enable early detection. If women or their partners and families are able to raise early warnings about potential signs and symptoms of pre-eclampsia in pregnancy, birth and the postnatal period, they may be able to receive appropriate intervention to prevent onset of severe pre-eclampsia from developing. Early warning signs of severe pre-eclampsia, which can deteriorate very quickly, include specific physical symptoms such as severe headaches that does not resolve with simple painkillers, problems with vision, such as blurring or flashing before the eyes, severe pain just below the ribs, heartburn that does not resolve with antacids, rapidly increasing swelling of the face, hands or feet and feeling very unwell [9].

Currently there are a limited number of strategies to help service users identify and seek help concerning a sudden deterioration in health, including pre-eclampsia. One exception is the NICE postnatal care quality standard [10] which recommends that all women are offered information on signs and symptoms of serious health problems within 24 h of the birth relevant to their own health and the health of their babies. This information should include prompts to enable women, partners and families to recognise serious health problems and when to seek urgent medical care [10]. A recent report which looked at implementation of the NICE quality standards on postnatal care found that 47% of mothers were unaware of signs of health deterioration in the postnatal period, despite the recommendations, and that around a quarter of women questioned were aware of signs and symptoms of health deterioration [11].

There is a limited amount of research regarding pregnant and childbearing women’s involvement with promoting their own safety. However, in UK maternity care there is increasing evidence that a number of women and their families do not always feel listened to or receive an appropriate response from healthcare professionals [12–14]. For example, a [12] highlighted that concerns of women were not being listened to. It showed that out of a random sample of over 23,000 women who gave birth in February 2013, 19% felt their concerns during birth and labour were not taken seriously.

Another example cited from a recent report of the UK National Perinatal Epidemiology Unit (NPEU) found that of the women whose babies died before labour, only 57% felt listened to or their concerns taken seriously. Of the women whose babies died during labour, a quarter felt that staff communicated poorly, almost half did not feel listened to and around 10% reported rarely or never having trust or confidence in the staff caring for them during labour and birth. The most common finding among women and partners whose babies died before labour was that 72% recognised that something was wrong with changes in their babies’ normal pattern of movement. When they were asked about raising their concerns to health care professionals, a third of women reported contacting a clinician straight away and 46% within 24 h, but only 57% felt listened to at this time and 39% felt confident with decisions made [14].

Patient involvement in safety of their own healthcare is an emerging field internationally. It has been highlighted by the World Health Organisation(WHO) who recognise that patients and carers are at the very centre of the quest to improve healthcare safety [15]. The Council of Europe and the World Alliance of Patient Safety have also recommended that patients should be involved in reporting of incidents and safety management [16, 17].

This narrative synthesis examined the extent to which the available literature could inform a greater understanding of the experiences of women and their families who raised an early warning about potential symptoms and signs of pre-eclampsia, and the organisational response. The research question the review addressed was:

*“What are the factors that affect women and their families speaking up with early warning signs and symptoms of pre-eclampsia and what are the factors affecting health professionals’ response?”*

## Methods

To develop a focused question which included women’s, their families’ and clinicians’ perspectives and to facilitate the literature search, the SPICE (Setting, Perspective, Intervention, Comparison, Evaluation) Framework was used [18]. The SPICE Framework is adapted from the PICO (Patient problem, Intervention, Comparison and Outcome) Framework and was more appropriate for this study as it enabled inclusion of all the perspectives of interest. See Table 1 includes the search and selection strategy for further details.

**Table 1:** SPICE framework search terms

SPICE acronym	Keywords	Examples of alternative words
Setting	Health Care Systems Caring for women with pre-eclampsia	Hospital, community care, accident and emergency, maternity ward, antenatal clinic, postnatal Clinic, labour Ward, delivery Suite, birth centre, alongside midwifey units, community clinic, GP surgery
Perspective	Pregnant women and their families and health care staff	Antenatal, postnatal, pregnancy, labour, birth, obstetric, childbirth/midwives, obstetricians, managers, maternity support workers, partners, health care workers, women
Intervention	Speaking up about pre-eclampsia/Patient Participation in safety	Patient involvement, patient collaboration, patient partnership, patient centred care, decision making escalation of care, early warning systems, signs and symptoms, organisation, management, speaking up, seeking help, help seeking behaviour, rapid response,
Comparison	Health Care staff response	Communication, team work, listening, nurse patient relations, midwife patient relations
Evaluation	Women/families/health care staff response	Perceptions, thoughts, attitudes, behaviours

A narrative synthesis approach was selected as it produces a systematic, transparent approach, with guidance on enhancing trustworthiness [19–22]. The approach aims to produce a textual, narrative understanding of findings from included studies conducted in different settings and contexts [23].

The UK Economic and Social Research Council (ESRC) methods programme guidance was used to reduce risk of bias [20]. It includes a general framework or toolbox of four components namely; developing a theory, delivering a primary synthesis of findings of included studies, exploring relationships in the data and assessing the robustness of the synthesis. For the purposes of this review we used tabulation and thematic analysis to analyse the data. Thematic analysis incorporates aspects of the grounded theory approach which is the process of coding, sorting and organising data, but it does not included theoretical sampling ([24] p 265); [25].

Each paper was allocated a number. Other table headings included author, country, method, sample type and size and key findings, (which also took into account the authors’ discussion points) and a quality score for each paper (Table 2). Themes were extracted and adjusted, merged or excluded using the principle of the constant comparison methods and “one sheet of paper” (OSOP) method [25, 26] by the first reviewer WC with full text papers (Table 2). Contradictions and relations between the themes were explored before a final list of themes and subthemes were agreed by discussion and consensus of all of the reviewers using the OSOP method to visually map out themes and subthemes [26]. (Table 2).

**Table 2:** Characteristic of included studies

Paper number	Reference and country	Aim and setting	Methods	Participants	Relevant findings	Quality scores
1	Brewer et al, 2015 [31] USA	To evaluate the extent to which The Pre-eclampsia Registry responded to narrative inquiries and to ascertain the depth of information related to patient education. Online USA.	Online open ended questionnaire with free text format included for one question about patient education. Retrospective questionnaire.	807 participants, 301 provided a response for a total of 355 pregnancies.	Additional information for women with pre-eclampsia was identified by 241 participants: Themes identified included: • Symptoms • Definition of preeclampsia • Improved provider communication • Risk factors for preeclampsia • Postpartum preeclampsia • Closer monitoring • Psychological support • Complications • Dietary concerns	5
2	Harris et al, 2014 [39] UK	To investigate the potential psychological impact of providing pregnant women with formal risk information for an antenatal screening test for pre-eclampsia. One London NHS trust.	Cross-sectional semistructured interview study of women who had first trimester preeclampsia screening test. Retrospective interviews at 16 weeks gestation.	15 primigravida women, who had high risk results and 5 with low risk results at 12 week pre-eclampsia screening.	Two types of coping typologies regarding risk information for preeclampsia; “*Danger Managers*” who were focused on risk that pre-eclampsia posed to them and exhibited information seeking, positive behaviour changes and cognitive reappraisal coping mechanisms. *Fear Managers*” who had an external sense of control and focused on the risk that pre-eclampsia posed to the foetus and exhibited avoidance coping mechanisms. 3 others themes emerged, medicalising pregnancy, embracing technology and acceptability.	4.5
3	You et al, 2012 [36, 37] USA	To explore the extent to which pregnant women understand the symptoms and potential complications related to pre-eclampsia and to determine the factors that are associated with better understanding. A university clinic in USA	Face to face survey with one open ended question with free text.	Convenience sample of 112 women recruited between 18 and 40 weeks gestation. 110 completed survey.	The survey identified a poor understanding of preeclampsia with a knowledge deficit. Factors associated with a greater understanding of preeclampsia were higher literacy, multiparty, history of preeclampsia, a receipt of information about preeclampsia from a clinician or another source.	5
4	Vasconcelos de Azevedo et al, 2011 [38] Brazil	To understand the meaning of preeclampsia for pregnant and postpartum women 5and health care professionals Antenatal clinic and admissions unit of a public maternity hospital	Word association test and semi structured interviews.	51 pregnant women, 10 postpartum women, 87 health professionals completed word association test. 18 women, 2 postpartum women and 20 health professional were interviewed.	Thematic categories based on word association test and the interview were created to help the data analysis. The results together demonstrate that pregnant and postpartum women had no information about preeclampsia. The meaning of preeclampsia to pregnant and postpartum women were fear, risk, care and late of information. For health professionals the meaning were care, fear, risk, high blood pressure, oedema and proteinuria.	4.5
5	Barlow et al, 2008 [38] UK	To document women’s experience of admission to hospital with a pregnancy related complication, hypertension from their own perspective One UK Maternity inpatient ward	Qualitative descriptive study semi structured contemporaneous interviews with women after sudden and unexpected admission with high blood pressure, and for some additional symptoms such as proteinuria and or oedema.	12 women, two with diagnosis of pre-eclampsia at time of interview.	*Search for meaning* *:* 7 women had not noticed signs and symptoms and some felt frauds being admitted. 5 women had noticed not feeling well, reduced fetal movements and two had had previous pre-eclampsia and were anxious and uncertain. *Attribution to causality* *:* Some felt they had tried to relieve stress in their lives, so could not understand why their blood pressure had been raised, others described stressful events and felt this may have contributed to admission. *Information needs* *:* Women valued being told the truth about their care pathway with diagnosis but some women felt they were not given enough information and were reluctant to ask staff questions. They reported being anxious and scared. Inconsistent information from different staff members was noticed. *Social factors* *:* All women felt it important to have the support of their partner/husband and other family members. Seeing women go the delivery suite and to return with a healthy baby was reassuring whereas seeing women return for a caesarean with catheters and drips was seen as “scary”.	5
6	Kalim et al 2009 [32] Bangladesh	To assess differences in knowledge and care seeing behaviour in two districts of Bangladesh. Jessore, a high performing district of the country with higher literacy levels and lower maternal mortality ratio in comparison to a lower performing district of the country, Sylhet.	Mixed qualitative methods including free listing, rating exercises, hypothetical case scenarios and in depth interviews exploring the most commonly perceived complications, their relative perceived severity, knowledge of about signs and symptoms, care seeking behaviours related to PPH and eclampsia. Retrospective interviews at unknown time limit after event.	118 women in total partook in studies, 40 regarding danger signs and care seeking for preeclampsia.	For women in low and high performing districts performing districts identified both PPH and eclampsia as life threating complications. *Understanding and knowledge*; In both districts women appeared to have a basic understanding of how to treat complications and where and were to take women for treatment, however in real life case studies there were major differences between their understanding to the conditions and care seeking behaviours in response to both PPH and eclampsia which could contribute to the high rate of maternal deaths associated with both conditions. *Social and economic disparities affecting help seeking behaviour*; There were differences in care seeing practices in the two districts possibly reflecting social cultural differences, disparities in economic and educational opportunity and discrimination in the availability of care.	4
7	Lima de Souza et al 2007 [33] Brazil	To analyse maternal experiences of preeclampsia pregnancy with premature birth at a neonatal intensive care unit. State Hospital specialising in high risk pregnancies Brazil.	A qualitative study using focus group technique of women who had experienced pre-eclampsia with a premature birth. Retrospective focus groups whilst babies were still inpatients.	28 women who had experienced preeclampsia in pregnancy with a premature birth.	Themes included information on preeclampsia during prenatal care, experiences with a child in NICU, mother’s perception of NICU professional attitudes. *Information about pre-eclampsia;* it emerged from interview that women were unaware of pre-eclampsia which may have contributed to deficient preventative care and even to early hospitalisation. They only became aware after hospitalisation or by imminent premature delivery. Women feared their death or of losing their child. *Mothers experiences with a child in NICU*; First visit was often associated with shock, sadness and despair. During NICU daily routine difficulties were reported on not being able to hold child and seeing intensive treatments. Conflicts arose between home and hospital activities. Women also discussed joy of bonding with the child when first held their babies and when phototherapy and IV tubes were removed. *Mothers perceptions of NICU professional attitudes*; Difficulties were identified regarding for caring the child in the neonatal care unit accentuated by communication flows between health professionals and users.	5
8	Macgillivray et al 2004 [34] Jamaica	To assess the efficacy and acceptability of a patient held pictorial card aimed at raising awareness and appropriate health seeking behaviour response to prodromal symptoms of imminent eclampsia. Antenatal clinics in Jamaica.	Survey and contemporaneous and retrospective unstructured face to face interviews with staff and eclampsia cases postnatally. Time scale not given when interviews took place.	192 mothers were surveyed before distribution of maternal pictorial card with preeclampsia symptoms, and 134 after. 3 women were interviewed who had eclampsia after card distribution. 18 health care workers were interviewed in five antenatal clinics and obstetric team in a hospital.	Survey showed a mother’s awareness and response to symptoms improved significantly with use of pictorial information cards, posters and education of signs and symptoms of pre-eclampsia and there was a significant drop in eclampsia incidence. Post education programme there were 3 cases of eclampsia noted: *Case 1* had not received the card at her antenatal clinic and had not seen a poster. *Case 2* had a card and recognised the symptoms but went to her community health neighbour next door, delayed going to hospital and convulsed. *Case 3* was a young teenager who reported symptoms to the high risk clinic but was told to bed rest and return again in 1 week. At the time the condition of the referral hospital were overcrowded. Interviews with health care workers identified that they felt the card had enabled mothers to recognise symptoms that should be acted upon and had the unexpected benefit of giving a focus for discussion when the health care workers saw mothers in the antenatal clinic as well as improving their own knowledge of when to act.	4.5
9	Harrison et al, 2003 [40] Canada	To examine women’s experiences of and satisfaction with their involvement with health care decisions during a high risk pregnancy. A Western Canadian City.	In depth open ended semi structured interviews one month after birth with women who had experienced hypertension or threatened preterm delivery.	47 women; 16 women received in home care through a community programme, 15 hospitalised care and 16 women with in home care for index pregnancy and in hospital management of a previous pregnancy. 26 women had pregnancies threated by preterm delivery, 17 had hypertension and 4 had hypertension *and* preterm delivery.	Women felt an increased feeling of responsibility for the health of their baby and themselves. They exhibited two approaches to decision making; active partners; and passive involvement. *Women who wanted active involvement;* achieved it through one of 3 processes; struggling for, negotiating or being encouraged. *Women who wanted more passive involvement;* and women facing health crisis used the process of trusting the experts of nurses and physician. Women were satisfied if the care from the health professional was congruent with how they wanted to be involved in decision making.	5
10	Kidner et al, 2004 [41] USA	To describe the experience of mothers whose pregnancies were complicated with HELLP syndrome and to determine if such experiences could be clustered by common themes from which a model could emerge. USA, home telephone interviews in urban and rural settings.	Descriptive home telephone interview qualitative study of survivors of HELLP syndrome Retrospective interviews at 15 months to 13 years post delivery, with 2 years being the mean.	9 self-selected survivors of HELLP syndrome.	Participants expressed a loss of control and now knowing. 5 themes were identified; premonition, symptoms, betrayal, whirlwind and loss. *Premonition*; Just feeling something was not right. *Symptoms*; symptoms described as back pain, fatigue, not feeling well, shortness of breath, abdominal. Pain, vomiting, severe upper quadrant pain. *Betrayal*; women reported being led astray and deceived and having their concerns viewed as worthless. They reported a sense of betrayal for trusted women, health care providers and their own bodies. *Whirlwind*; with recognition and diagnosis of HELLP syndrome physicians initiated an intensive whirlwind of activity to save mother and baby. *Loss*; loss and grief caused by HELLP syndrome delivery that was so different form the expected pregnancy outcome. Emotions expressed were fear of death, frustration, anger and guilt.	5

### Quality appraisal

There is considerable debate on whether or not concepts such as validity and reliability apply to qualitative research and if so how these could be assessed [27]. Cochrane methodological guidance for qualitative methods involves (i) filtering against minimum criteria, involving adequacy of reporting detail on the data sampling, collection and analysis, (ii) commenting on technical rigour of the study elements indicating methodological soundness and (iii) paradigmatic sufficiency, referring to researchers’ responsiveness to data and theoretical consistency [27]. However, some authors consider that formal appraisals of quality may exclude some studies ranked as ‘lower’ in terms of technical markers of quality, but ratings may not be sufficient to invalidate the findings [28, 29]. To maximise the inclusion and contribution of a wide number of studies, a low quality threshold was set using Dixon-Woods et al’s five point checklist for quality [30]. Research was included regardless of quality due to the difficulty of assessing this among of studies which used a wide range of methods. The overall quality of papers was high (Table 2) supporting the robustness of the synthesis and findings. The lowest score, which was allocated to one paper, was 3.5/5 and five out of the 10 papers scored 5/5.

### Search and selection strategy

Initially a systematic search of relevant literature was completed. Search strategies were constructed using key headings in SPICE to guide the review. Words from the research question which were adapted depending on the database. For example, a combination of free text and the database’s own subject headings were used. The following electronic data bases were searched; Medline, CINAHL, HMIC, PsycINFO, Embase, BNI, ASSIA, Scopus, Maternity and Infant Care, Web of Science, Google Scholar, Cochrane, JBI and IBSS with the support of an Information Service Consultant from January 1980 to April 2016.

A typical strategy for this search was: (“Pregnant women” or “pregnant women and their families” or patients or childbirth or midwi* or obstetrician* or hospital* or manager* or “maternity support worker*” or childbirth or “health care worker*” or labour or families or partners) AND (preeclampsia or “pregnancy complications” or “obstetric complications” or “pregnancy induced hypertension” or “high risk pregnancy” or eclampsia or “maternal mortality” or “maternal morbidity” or deterioration) AND (“patient participation in safety” or “patient collaboration” or “escalation of care” or “patient centred care” or “patient involvement” or “patient choice” or “nurse patient relations” or “doctor patient relations” or “patient empowerment” or “early warning systems” or signs or symptoms or “speaking up” or “seeking help” or “help seeking behaviour”) AND (“women’s experiences” or “staff response*” or perception* or attitude* or behaviour*).

Other methods included hand searching references lists and citation tracking.

### Inclusion criteria

English language studies were selected that used a range of qualitative approaches to capture evidence of the experiences of women and individuals in their immediate social network, such as their partner or other close family member, of pre-eclampsia and eclampsia.

### Exclusion criteria

Quantitative studies were excluded as were mixed method studies if it was not possible to retrieve free text data. Non English language papers and grey literature was excluded.

## Results

**Fig. 1:**
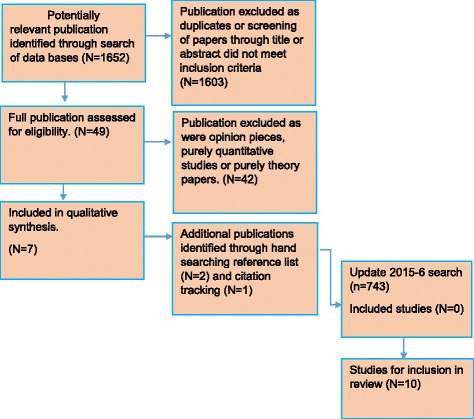
PRISMA diagram to show flow of articles selected

A total of 2395 records, titles and abstracts were screened for inclusion in the review, of which 2346 were excluded as they were either duplicates or did not meet the inclusion criteria, leaving 49 articles remaining. Of these, 42 were excluded once the full article was assessed for eligibility, as they were either purely quantitative papers, mixed method studies where the results could not be separated or opinion pieces, leaving seven articles for inclusion in the review overall. A hand search of the reference lists identified two further studies and another was identified through citation tracking. Thus the total number of studies selected for the narrative synthesis was 10 (Fig. 1).

### Data extraction and synthesis

Data extraction and synthesis are described in the methods section.

Table 2 summarises findings from the 10 papers. Three studies were from the United States, two from the United Kingdom, one from Canada, one from Bangladesh, two from Brazil and one from Jamaica. Topics covered by the studies are discussed in the narrative synthesis. Total number of participants involved in each study are also summarised in Table 2.

Table 3 summarises themes and subthemes identified during the narrative synthesis using the OSOP method [26]. By using allocated paper numbers (Table 2), Table 3 shows how data were retrieved for each sub-theme.

**Table 3:** Summary Table of Themes and sub-themes

Theme	Subtheme including paper where data were retrieved
Theme 1: Women’s knowledge and understanding of preeclampsia	Knowledge (1, 3–8)
	Absence or not recognising signs and symptoms (2, 5, 8–10)
	Range of information needs (1–3, 5, 8, 9)
Theme 2: Factors affecting help seeking behaviour from perspectives of women and their families’	Emotions affecting help-seeking (2, 4, 5, 7, 10)
	Social, cultural and economic disparities (6, 9)
	Social networks influencing help-seeking (5, 6, 10)
Theme 3 : Factors affecting staff response	Practitioner-client communications and relationship (1, 4, 5)
	Not being taken seriously (4, 8, 10)

### Narrative synthesis

#### Theme 1: Women’s understanding and knowledge of pre-eclampsia

**Subtheme 1: knowledge**

A common finding was that many of the women who participated in the included studies did not have an understanding of pre-eclampsia or its implications for their own or their babies’ health. Many were also unaware of signs and symptoms of pre-eclampsia until after their initial diagnosis [31–37]. It was unclear if these women had any specific symptoms or signs and their lack of knowledge meant they did not appreciate their significance of these, or if they did not actually have any early warning of onset.

A lack of knowledge of pre-eclampsia among women who developed the disorder was reported from studies from several countries, including studies from Brazil, Bangladesh, USA, and Jamaica. For example in Brazil, all of the women interviewed in [35] study of the meaning of pre-eclampsia reported that they received almost no information about the disorder during their doctor’s appointment or during the period that they were hospitalised. Women reported that they wanted to know more about the development of pre-eclampsia, how to avoid it, and the consequences of the disorder. A second study from Brazil reflected these findings in cases where women who had had a preterm birth as a consequence of pre-eclampsia were unaware of the pre-onset of the disorder. It was postulated that this may have contributed to deficient preventative care and to early hospitalisation caused by the severity of their condition [33].

Similar findings were found in the UK, for example, women reported that admission to hospital had been sudden and unexpected following a routine antenatal clinic appointment due to high blood pressure and hypertension, and some had additional symptoms such as proteinuria and oedema [38]. This was also reported in studies from Brazil and the USA [33, 36, 37] further highlighting women’s limited awareness of the signs and symptoms of pre-eclampsia.

Although most included studies highlighted a lack of understanding of pre-eclampsia or awareness of possible symptoms and signs, previous personal experience of pre-eclampsia was an important influencing factor. Barlow et al [38] in a study from UK found that two out of the 12 womeninterviewed had pre-eclampsia in previous pregnancies and as a consequence were aware of symptoms and signs in the index pregnancy, which led to them to self-refer for medical assessment and hospital admission by the process of early recognition and seeking professional help. The impact of having had pre-eclampsia in a previous pregnancy was also identified in the study in Brazil, however this knowledge resulted in a subsequent fear of the disease. The study did not specify if previous experiences and knowledge had altered women’s help seeking behaviour [35]. The quote below from a woman who had had pre-eclampsia previously highlights the fear the women experienced;

“*I am afraid, I am afraid of having it again (pre-eclampsia)…it’s because after the pregnancy I didn’t have any high blood pressure problem, but then now on my second pregnancy I’m having it all over again (high blood pressure)…”* ([35] p 184).

**Subtheme 2: absence of or not recognising signs and symptoms**

The synthesis identified that women’s lack of knowledge may prevent them from seeking timely and appropriate medical support due to a lack of awareness of pre-eclampsia and its implications [34, 38–40]. However another finding includes women who did not notice the onset of symptoms and signs of pre-eclampsia which may have ‘prompted’ them to seek help. Barlow et al [38] found that seven of the 12 women in their study did not notice symptoms or signs at all and consequently felt ‘frauds’ when they were admitted as an in-patient and took up a hospital bed as the following quote illustrates.

*I was in shock and upset that I had to come in. The only thing I know is that I’ve got high blood pressure and protein and something in blood. It’s to do with pre-eclampsia, but I didn’t understand what this is cos I’m fine in myself and the baby’s fine… and you’re thinking why can’t I go home?* ([38] p161)

Some women had difficulty differentiating between common pregnancy symptoms such as nausea, vomiting, heartburn, oedema and the early symptoms and signs of pre-eclampsia. Clinical staff also had similar problems [38, 41]. In two studies, women reported feeling that ‘something wasn’t right’, with fatigue, not feeling well, shortness of breath, abdominal pain, nausea and vomiting, and severe right upper quadrant pain and reduced fetal movements all reported as symptoms and signs experienced by women questioned in the studies [38, 41]. The quote below was from a women who experienced symptoms and signs of pre-eclampsia but did not seek help.

*“I had everyone in the world, who was wiser than me, telling me that this was heartburn, that this was reflux, that this was stomach problems, that this was tensions I actually let the pain go unchecked”* ([41] p 48).

**Subtheme 3: range of information needs**

The studies selected for synthesis generated little insight into what information women needed, or when or how they would like to receive information. In four of the included studies women would have liked more information on pre-eclampsia [31, 34, 36–38]. In the study from Jamaica, there was a marked decline in the incidence of eclampsia 6 months after women were offered information on what symptoms and signs to look out for, using pictorial cards, posters and antenatal education [34]. The authors’ considered that the intervention resulted in an improvement in the women’s awareness of the importance of action concerning particular symptoms and of response to clinical advice in general. It also had the unexpected benefit of providing a focus for discussion when the healthcare workers saw the women in the antenatal clinic. The study also appeared to have resulted in an increased awareness among the health care professionals of the prodromal symptoms of eclampsia reminding midwives of important symptoms and signs to look out for and treat [34].

Of note, is that not all women in this study were supportive of use of the pictorial card. Younger women in particular felt the card was not relevant to them they as it depicted an older woman [34].

Only one study reported that women searched the internet for more information regarding pre-eclampsia [39]. This was generally after women had a high risk screening test result for pre-eclampsia. Some of the women used the information to consider how pre-eclampsia would affect them and wanted to know how to prevent it from developing whereas others were more selective of the information.

One UK study which had conflicting results explored the psychological impact of providing women attending two maternity hospitals with risk screening information for pre-eclampsia in the first trimester of pregnancy, with conflicting results [39]. Although the majority of the women felt that if they knew they were at increased risk of pre-eclampsia they would have the advantage of being more likely to recognise the onset of the disoreder; a minority questioned the usefulness of providing information for a condition that they perceived had no treatment and would increase their anxiety without providing a clear benefit [39]. These findings supported those of the earlier study by Harrison et al [40] in Canada who found that some women struggled to get the information they needed to participate in decision making. Other women were satisfied with passive involvement in decision making and wanted to trust the experts, perceiving healthcare professionals as having current and specialised knowledge that would be beneficial for them and their expected infant as the following quote illustrates.

*“If Dr M told me I had to stand on my head every morning for 10 min, I would do it. I would just do what she would tell me to do..they (doctors)_ are a lot smarter than me.”* ([40] p112)

The amount of knowledge required was affected by women’s individual attitudes; an emerging finding was the impact of the women’s emotions on help-seeking once they noted symptoms and signs of pre-eclampsia. This is discussed in the theme below.

#### Theme 2: factors affecting help seeking behaviour from perspectives of women and their families’

**Subtheme 1 emotions affecting help-seeking**

Feelings and emotions were also found to alter women’s help seeking behaviour and the amount of information they required. Fear was reported as affecting behaviour in five of the included studies [33, 35, 38, 39, 41]. Vasconcelos de Azevedo D et al. [35] found that women who were afraid of the disorder and were aware that they and their infants could be in danger as a consequence, knew they needed to seek urgent medical care. The women wanted to be listened to and needed to understand what was happening to them. Kidner [41] identified women experienced not only fear but also anger and women felt frustration during their efforts to seek relevant and timely information, as one woman explained.

In Barlow et al’s [38] study three women reported being anxious and scared; one woman had written a list of questions but was too upset to ask them. Another woman felt the doctors used big words or provided conflicting information as “*one says one thing and another says another”*. Here the perceived power imbalance between the health care professional who used complicated medical language affected the women’s ability to secure an appropriate response for their concerns.

**Subtheme 2: social, cultural and economic disparities**

Other factors that affected women’s and their families help seeking behaviour reflected social and economic disparities as reported in the study from Bangladesh [32]. In the ‘low performing’ Sylhet district (this is a term used to describe a district which has low literacy levels and a high maternal mortality ratio compared to other districts), family members first administered treatment in the home even after convulsions had started and only when these attempts failed did they consider hospital admission. This was in contrast to a ‘high performing’ district, Jessore, where family members called in medical assistance and transferred the woman to a health facility after the convulsing had started. Reasons proposed for these differences included cultural beliefs i.e. that eclampsia was associated with evil spirits and structural barriers i.e. not being able to locate transport to take the woman to a health facility [32]. One respondent in Syhlet said;

The study authors did not include quotes describing pre-eclampsia symptoms and signs but in three cases, the women’s family members brought blessed water from a huzur (spiritual healer) as they believed that the Dushi was the reason for the convulsions.

Other sociocultural differences were also identified in the same study e.g. Sylhet women were less educated making them more reliant on relatives, more conservative with a fear of male service providers possibly attending them in a health facility and physical barriers including greater travel distances between the woman’s home and the healthcare facility.

No other study specifically explored or compared impacts of social and economic disparities. In Canada, a woman’s level of education was not associated with negotiation of participation in their health care decisions or with their level of satisfaction with their involvement in decision making [40]. It is possible the USA, UK, Brazil and Jamaica, had socioeconomic disparities which did affect help seeking, however it was not possible from the studies synthesised to identify differences in comparing women/health care professionals accounts from these perspectives.

**Subtheme 3; social networks influencing help-seeking**

Three papers identified the influence of women’s social networks on help-seeking behaviour [32, 38, 41]. Although positive emotional support was recognised [38], the potentially harmful influence of friends and family was also cited i.e. some women received false reassurance after seeking advice from other women regarding symptoms and signs [41]. This is reflected in the advice provided by a relative of a woman from Jessore who had developed a headache prior to fitting.

#### Theme 3: factors affecting staff response

##### Subtheme 1: practitioner-client communications and relationships

There were limited findings on factors affecting healthcare professionals’ responses to women and their families’ presenting with self -diagnosed signs and symptoms of pre-eclampsia. Practitioner-client relationships were mentioned in some studies. In Brewer et al’s [31] online patient questionnaire in the USA, open ended responses were collected from the question *“Is there any other information about this pregnancy that would be helpful to you?* Improved provider communication was identified as one of the emergent themes from this question [31]. Desired areas for additional information included symptoms, definition of pre-eclampsia, improved provider communication, risk factors for pre-eclampsia, postpartum pre-eclampsia, closer monitoring, psychological support and complications and dietary concerns [31]. Vasconcelos de Azevedo [35] also identified a communication gap between women and health professionals with regard to their experiences of pre-eclampsia. For example, fear and risk were associated with the disorder for women, whereas for health care professionals their main focus was framed in terms of physical treatment.

Women were reported as finding the divergence of opinion about their symptoms and signs among health care professionals confusing and conflicting [38]. Barlow et al’s [38] research highlighted the need for healthcare professionals to be consistent as crucial to enabling women’s positive experiences of care. One woman noted that day and night shift professionals often provided inconsistent information. The quote below illustrates this issue:

*“I am fed up with it, cos they tell you different things…when I got brought in, they says you’ll be in for a fortnight and you’ll probably have the baby. And next breath….you’ll probably have the baby in 2–3 days. Then everything is on a level, and they say, we want to keep you until 36 weeks now”* ([38] p163)*.*

Again there was little to inform why inconsistent information was offered to women, but factors such as the model of care provision, the workload of wards or individual health care staff may have influenced the provision of information.

**Subtheme 2: Not being taken seriously**

In three papers some of the women questioned reported symptoms and signs of pre-eclampsia but did not receive what they perceived to be an appropriate response from the healthcare professionals [34, 41]. An example of this was a young teenager who reported her symptoms to healthcare professionals during attendance at a high risk pregnancy clinic, but was advised to take bed rest and return to the clinic after 1 week. She later developed eclampsia [34]. Nine women from the USA who had had HELLP syndrome with significant symptoms-did not automatically seek medical assistance from healthcare providers. Some asked other older, perceived to be wiser, women about their symptoms, who reassured them that they were normal. Others had then escalated their concerns to health care professionals, but again were told not to worry as their symptoms were perceived as normal or resembled common pregnancy ailments, and advised not to worry [41]. After diagnosis the same symptoms and signs the women had reported were acknowledged as important and dangerous indicators of HELLP syndrome. This left women feeling betrayed after the preliminary delayed recognition and diagnosis. Additionally once admitted, some women felt their symptoms were devalued and treated as normal characteristics of pregnancy [41], as the following quote illustrates.

Here the woman reported that the healthcare professional not only ignored her attempts to raise awareness of her symptoms, but belittled her, which had the effect of preventing the woman voicing her health care concerns again.

Although specific examples were not provided, Vanconcelos de Azevedo et al’s [35] study highlighted that staff did not listen carefully to the women to understand how they were feeling and usually only considered their own perceptions.

Only one study investigated reasons why healthcare professionals did not respond appropriately to a woman seeking advice on regarding her symptoms. In MacGillivray’s study, where a teenager was told to take bed rest and return again after 1 week, the doctor made his decision as the maternity ward was running over capacity [34].

## Discussion

Only 10 studies were eligible for inclusion in the narrative synthesis highlighting the limited amount of research available in this area. Furthermore in these studies there was limited evidence of healthcare professionals’ perspectives. Despite this, from the evidence considered in this synthesis, three main themes were identified; women’s knowledge and understanding of pre-eclampsia and potential signs and symptoms of onset; factors affecting help seeking behaviour from the perspectives of women and their families’ and factors affecting staff response. The most dominant theme related to women’s lack of information and understanding of pre-eclampsia. Many wanted information on signs and symptoms, and experienced being offered conflicting information by healthcare professionals as disempowering. The synthesis also identified that some women did not develop noticeable “classic” symptoms or signs of pre-eclampsia or found it difficult to distinguish these from ‘normal’ pregnancy health. This is an important finding as it raises questions about the scope for women and families to contribute to this process given so many were unaware of signs and symptoms.

Many of the themes identified in the findings are supported by research in other health areas, for example that individuals need more information in order to become “the expert patient” [42], the belief being that by developing education programmes and creating expert patients, individuals are better able to take responsibility for their own self-management. However, expert patient research to date is often confined to individuals with chronic diseases such as diabetes [43]. There is little research exploring what are perceived as “normal” signs and symptoms of pregnancy and how best to differentiate between these and more serious symptoms and signs, which might indicate pre- eclampsia or eclampsia which is an acute medical emergency. important finding was that only one paper linked socioeconomic disparities with help seeking behaviour [32] and pre-eclampsia. Other studies have identified the significance of literacy levels for help seeking [44]. The authors of a Belgian study suggested that those women at greater risk of severe pre-eclampsia were asylum seekers who did not receive adequate antenatal care as they were unfamiliar with the maternity system of the host country [45]. In the UK, the findings of a recent perinatal mortality report were that women from non-white ethnic groups and women in the most deprived quintile had stillbirth and neonatal death rates twice those of white women and those resident in the least socio-economically deprived areas (Maternal, Newborn and Infant Clinical Outcome Review Programme (MBRRACE-UK) 2016). The lack of findings in this narrative synthesis suggests this is an understudied area.

Another important finding is from the four studies that identified the influence of a family member or partner on a woman’s help seeking behaviour [32, 34, 38, 41]. This ranged from generalised emotional support [38] to women seeking help from other women regarding signs and symptoms [41] and receiving false reassurance, to women actually being prevented from getting urgent medical care needed [32]. The opposite was found in a qualitative organisational case study as part of the Birthplace in England research. Of 58 women interviewed regarding an escalation of care requirement, 14 reported speaking up in situations they felt to be urgent. The women also identified their relatives or support partners playing an important role in helping them to speak up [13] and illustrated how they can act as safety buffers by voicing concerns and pre-empting failures in care [13].

At times healthcare professionals had difficulties differentiating what women reported as important and requiring urgent escalation of care. Although the research was limited some women described not having their health care concerns responded to appropriately in some situations. Help seeking behaviour among the women could also have been affected by their attitudes and emotions [35, 39–41]. In some cases emotions such as fear for their safety and frustration encouraged women to speak up [35, 41], whereas in other cases emotions such as anxiety and being scared overwhelmed their ability to ask questions [38]. In [38] paper the perceived power imbalance between the health care professional and woman and the medical language used affected women’s ability to secure an appropriate response to their concerns. The women’s emotions were also affected by the health care workers behaviour supporting Nadler’s [46] definition of help-seeking as “a three way interactive process that involves the recipient, the helper and the task or problem” [46]. Other studies also identified that women’s attitudes and characteristics themselves affected whether or not they wanted to seek help [39, 40] for example if they wanted to be passive or active in their own care pathways. These studies did not indicate how differing behaviour patterns affected speaking up about signs and symptoms of pre-eclampsia, but possibly those women who relied on the expertise of staff would be less likely to question a health professional’s decision to discount her concerns. These findings also challenge policy assumptions that all women want to actively contribute to their safety.

Thus the role of emotions when seeking help with symptoms and signs of pre-eclampsia cannot be isolated without understanding the complex sociocultural nature of help-seeking. Other authors have identified multiple variables of help-seeking with reference to symptom perception [47–51], but none have researched pregnant women and pre-eclampsia.

This systematic review and narrative synthesis has highlighted the paucity of research in this important area and raises a number of implications for future research. Further research is needed to establish what information is needed, and how and when women and their families would most benefit from it. Understanding women’s typologies; including those who want to be more involved in their care and those who want healthcare professionals to take responsibility, and how this affects their health seeking behaviour, warrants inquiry. This is an underexplored area and needs further investigation with respect to pre-eclampsia. A key finding of this review is that there is very little qualitative research available which explores how healthcare professionals’ responses affect women’s help-seeking with pre-eclampsia.

Implications for practice include health education to inform women and their families about symptoms and signs of pre-eclampsia/eclampsia, and raise awareness that some women may not develop noticeable signs and symptoms of pre-eclampsia or may find it difficult to distinguish these from ‘normal’ pregnancy characteristics. Healthcare professionals also need training and guidance on how and when to offer women information regarding pre-eclampsia and to be made aware that women’s individual attitudes and emotional needs can affect help-seeking. Staff training also needs to address the importance of socio-cultural factors in women’s self-monitoring, sense making of symptoms and signs and help seeking behaviours. Individual hospital guidelines and policies need to be developed accordingly, tailored to the needs of their local population. The new WHO antenatal guidelines recognised the importance of regular, high quality antenatal appointments which should include information on health promotion, and discussion of screening and diagnosis, and disease prevention [52].

### Limitations of the syntheses

One of the limitations of this synthesis was the dearth of research in this area, which resulted in the inclusion of only 10 studies, which had a wide range of aims and methodologies. Mixed method papers were only included if they reported free text comments although it was often unclear how researchers had analysed the free text. For example You et al. [36, 37] stated that the patients’ verbatim responses were recorded and independently rated as or correct or incorrect by their obstetrician. Women at different stages in their pregnancies were also included and the studies addressed a variety of research questions (some of which were quite different to the question asked for this synthesis) potentially shaping the nature of the findings. Capturing women’s views retrospectively and contemporaneously, or narratives gained through repeat interviews over time may also have impacted negatively on the synthesis.

There was no demographic or geographic restriction to selected studies, which were carried out in a number of countries with different health care systems, sources of funding and maternity care clinicians making it difficult to compare results. The study settings included upper, upper middle and lower income countries which also could have affected synthesis of the findings e.g. the ability of an individual to pay for healthcare is likely to impact on help seeking even when presenting with prodromal signs of pre-eclampsia. In addition transport and access issues will vary across the countries.

A further limitation is that any review will be subject to the research is available at the time and may be driven by the researcher’s agenda rather than patient informed priority [53].

By analysing free text the aim was to capture the user and staff voice. However, by limiting the inclusion criteria to qualitative studies and mixed method studies with free text the scope of review may have been narrowed.

## Conclusion

This narrative synthesis found a lack of knowledge and understanding about pre-eclampsia among women and their families’, and a lack of awareness of the symptoms and signs of onset of the disorder. Of importance for healthcare professionals is the finding that not all women present with ‘classic’ symptoms and signs of pre-eclampsia or can distinguish these from normal pregnancy characteristics, with potential psychological, emotional and socio-cultural influences also likely to influence an individual woman’s initial help-seeking behaviour.

Further research into women’s information needs with regards to pre-eclampsia which stresses the importance of attendance at routine antenatal contacts, as well as raising awareness among healthcare professionals that their responses to women who do speak up can impact on women’s help-seeking behaviours is needed.

## Abbreviations

CQC: Care quality commission
ESRC: The UK economic and social research council
HELLPP: Haemolysis, elevated liver enzymes and low platelet
JLA: James lind alliance
MBRRACE-UK: Maternal, newborn and infant clinical outcome review programme
NICE: National institute for health and care excellence
NPEU: National perinatal epidemiology unit
OSOP: One sheet of paper
PICO: Patient problem, intervention, comparison and outcome
RCM: Royal college of midwives
RCOG: Royal college of obstetricians and gynaecologist
SPICE: Setting, perspective, intervention, comparison, evaluation
UK: United Kingdom
USA: United States of America
WHO: World Health Organisation
